# Supplementary material for: Antisense Morpholino-Based In Vitro Correction of a Pseudoexon-Generating Variant in the SGCB Gene
Source: Int J Mol Sci. 2022 Aug 29;23(17):9817. doi: 10.3390/ijms23179817 (PMC9456520; doi:10.3390/ijms23179817)
Supplement: Supplementary file 1 [file ijms-23-09817-s001.zip › ijms-1773454-supplementary.pdf]

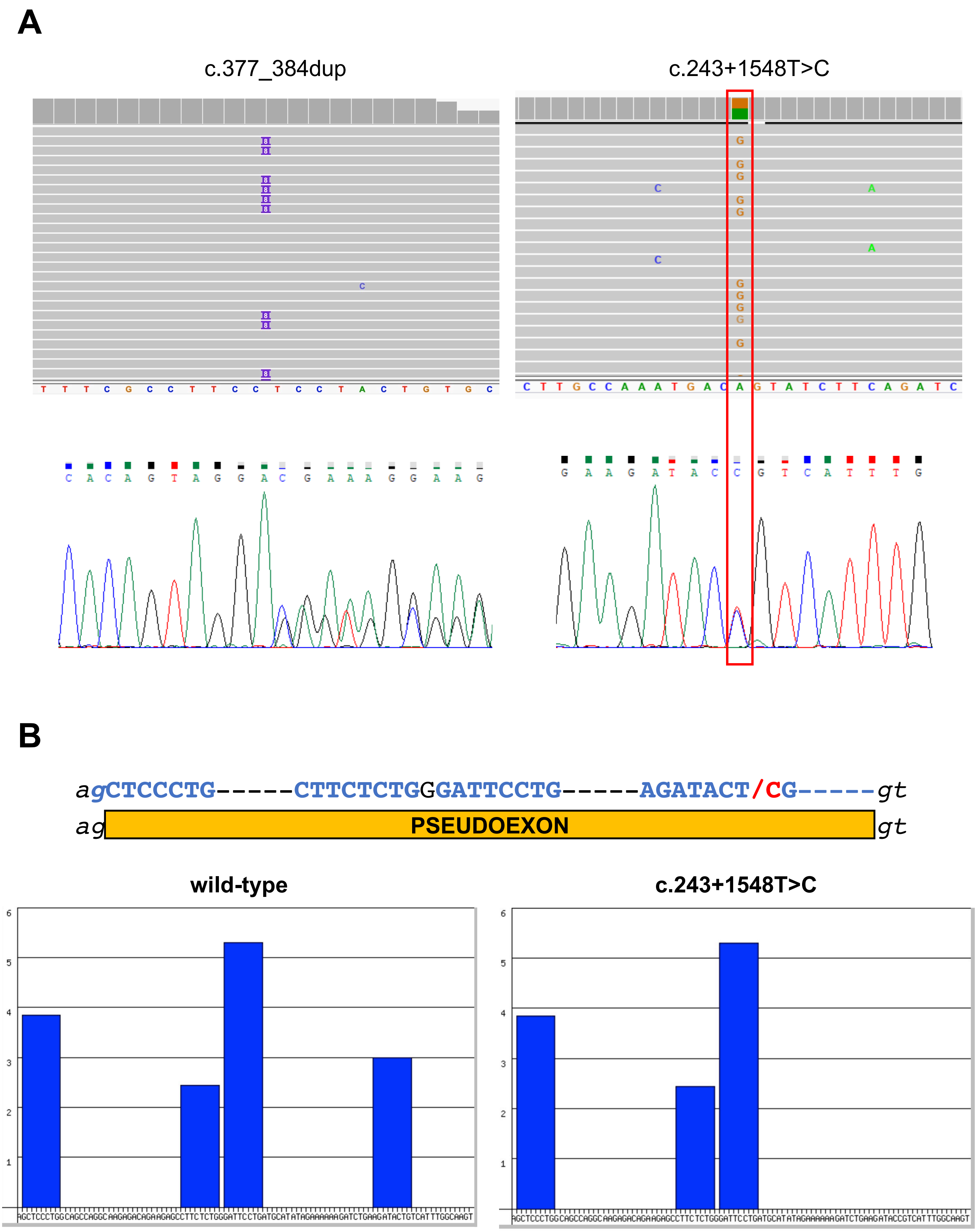

Figure S1 Genetics findings in our Patient. (**A**) Integrated Genomics Viewer (IGV) screenshots and sequence electropherograms displaying the *SGCB* variants detected in the Patient described in the manuscript. (**B**) ESE-finder 3.0 screenshots showing the prediction of the Serine And Arginine Rich Splicing Factor 2 (SRSF2) splicing factor binding sites (in blue in the sequence) in wild-type and mutated (c.243+1548T>C) sequence.

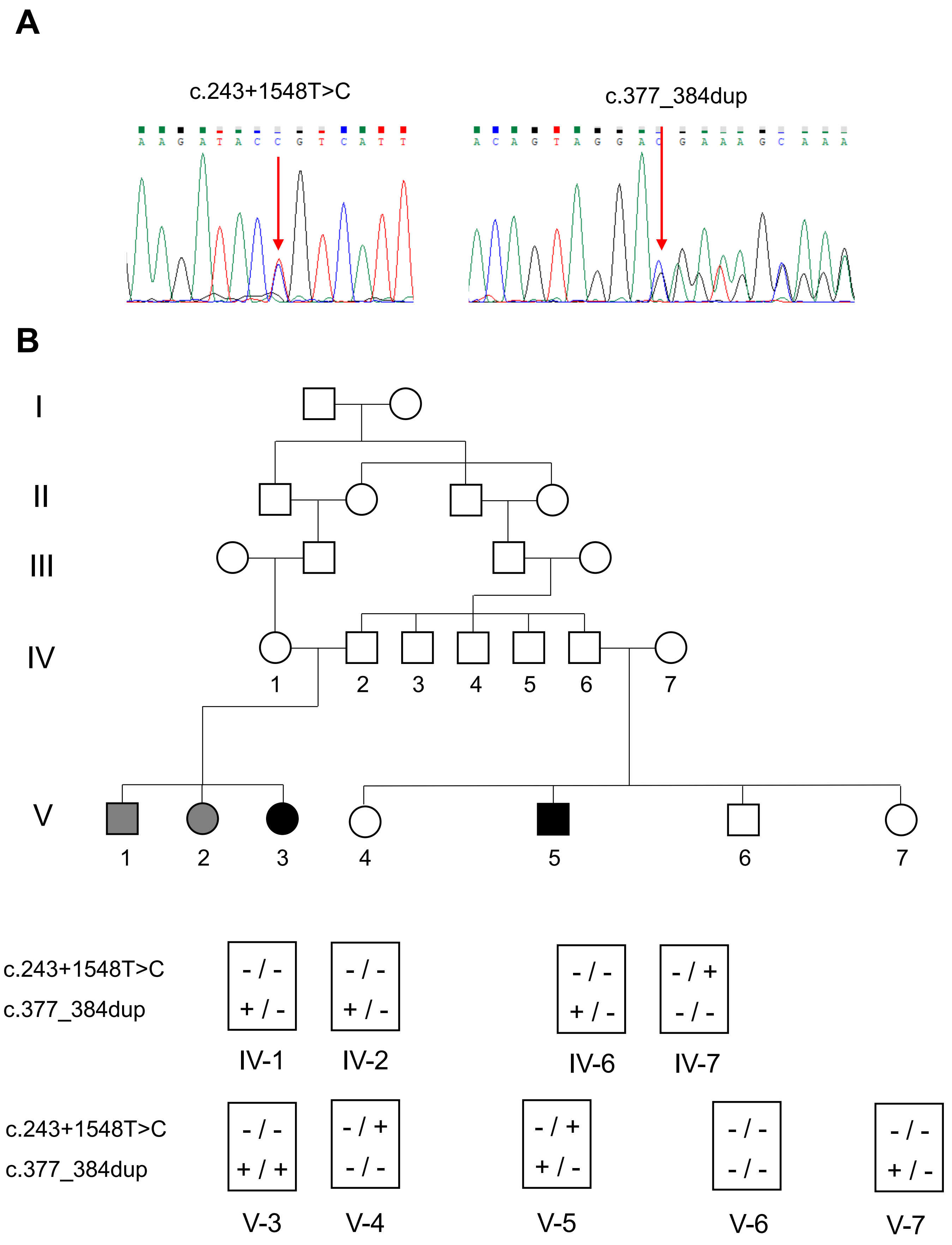

Figure S2 Genetic findings in a previously reported LGMDR4 patient. (**A**) Sequence electropherograms displaying the *SGCB* variants detected in the Patient V-A described in Barresi *et al.*, 2000. (**B**) Pedigree of the family described in Barresi *et al.*, 2000 now reporting the genotype of the *SGCB* c.243+1548T>C and c.377_384dup variants in available family members.

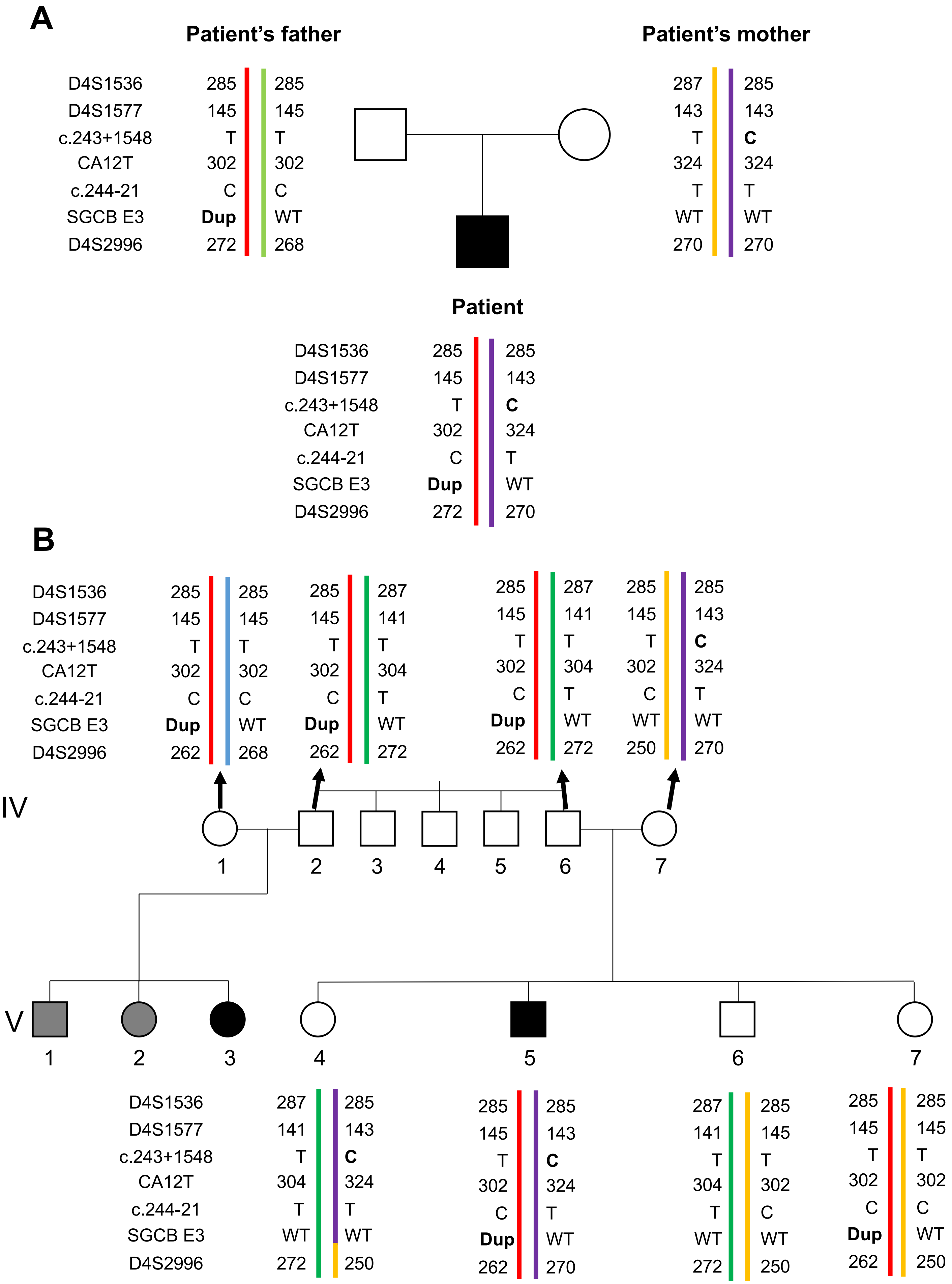

Figure S3 Microsatellite analysis around SGCB locus in the pedigrees described. Microsatellite markers analysis in the family described in the manuscript (**A**) and in the pedigree previously reported by Barresi *et al.*, 2000 (**B**). "Dup" indicates an allele harboring the c.377_384dup mutation.

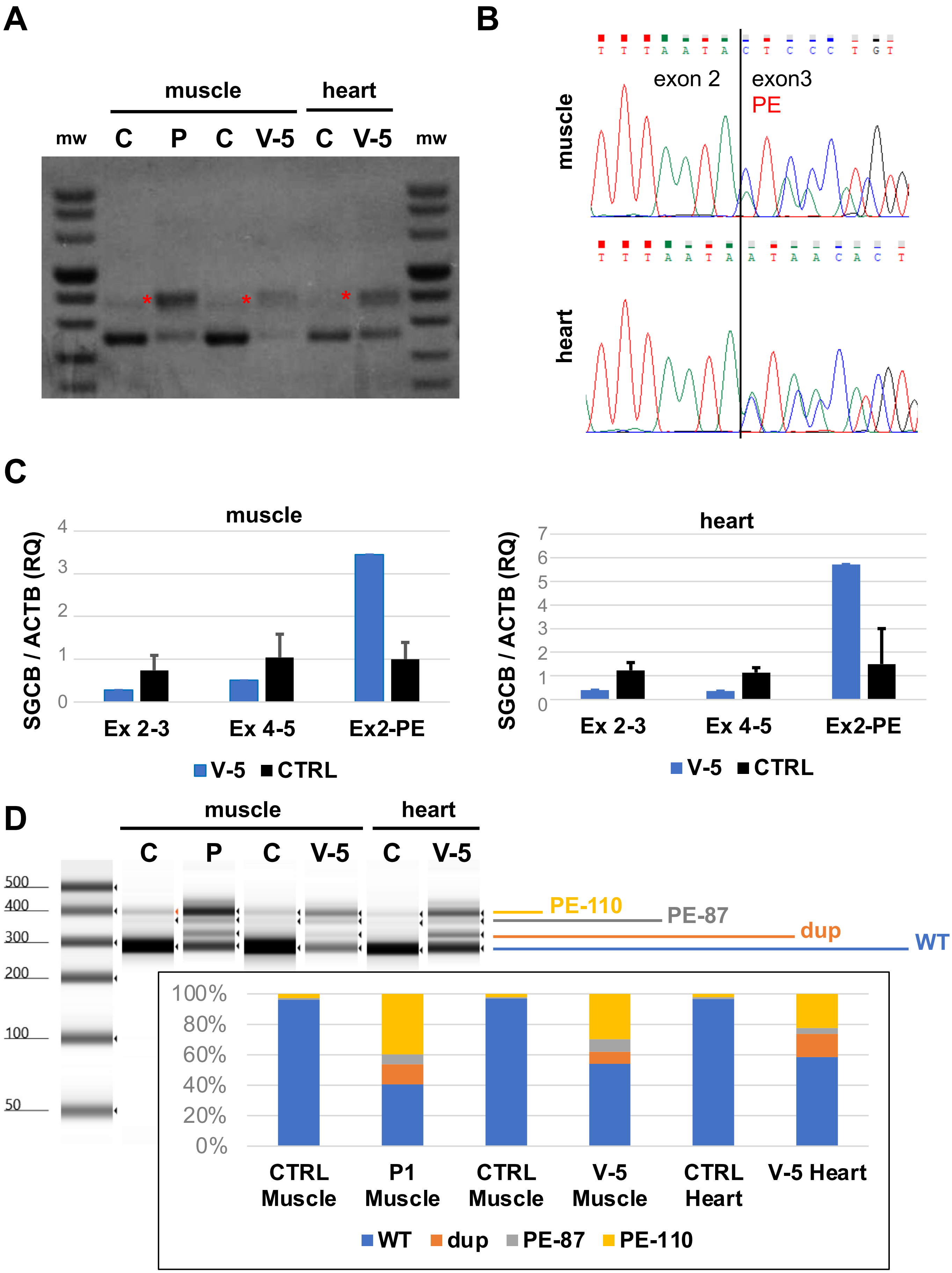

Figure S4 Transcript analysis of SGCB in a previously described LGMDR4 patient. (**A**) RT-PCR analysis of *SGCB* transcripts in tissues collected from the patient described in the manuscript (P), the previously-reported patient V-5 and controls (C). (**B**) Sequence electropherograms displaying the abnormal Exon2-PE junction in Patient V-5's tissues. (**C**) Quantitative RT-PCR experiments evaluating physiological (Ex2-Ex3, Ex4-Ex5) and abnormal (Ex2-PE) splicing junctions in Patient V-5's tissues and control biopsies (n=3). (**D**) Tape station analysis of RT-PCR amplicons showed in panel A documenting the presence of different levels of aberrant and physiological splicing products in patients' and control tissues.

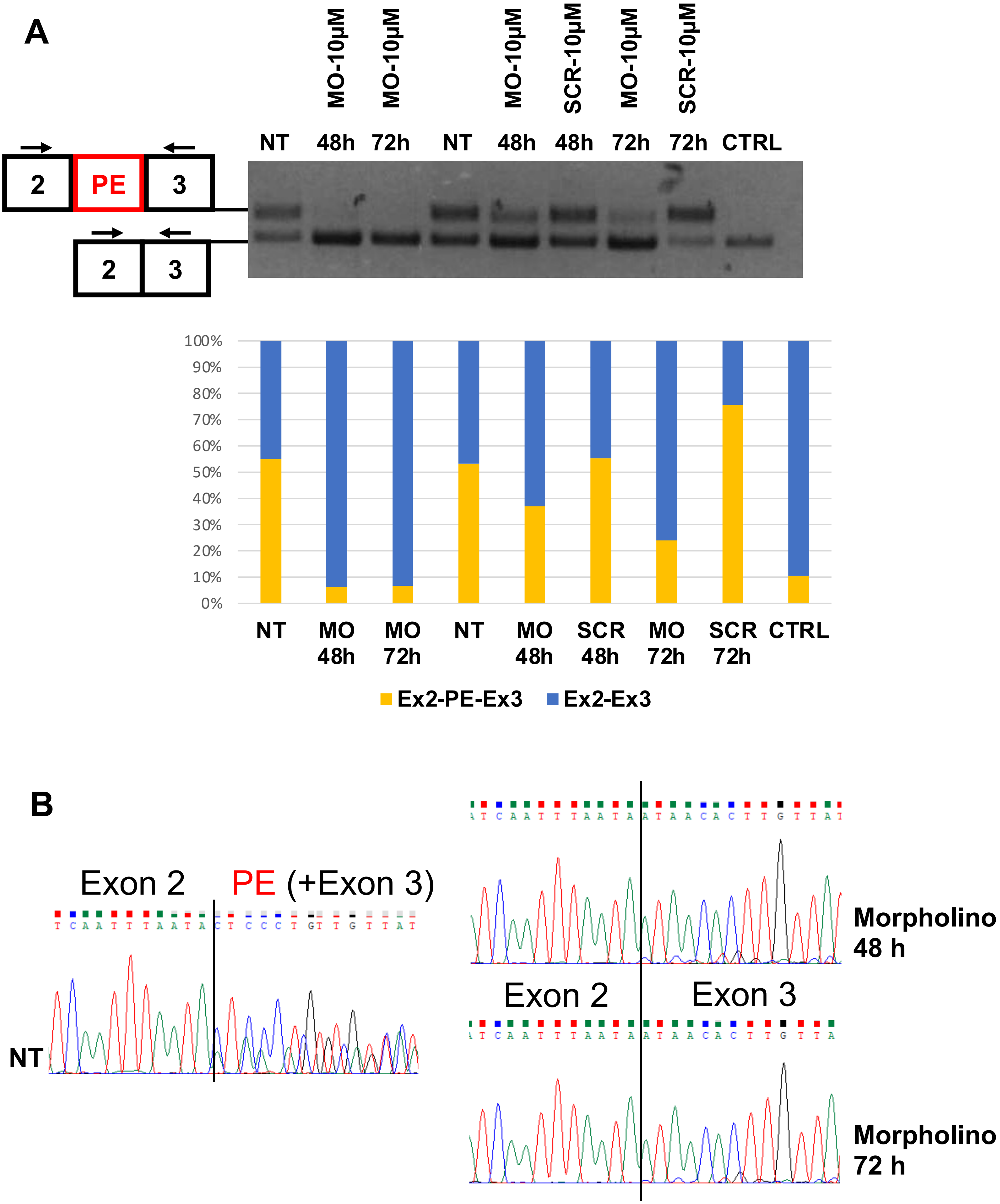

Figure S5 In vitro correction of splicing defect after Morpholino treatment. (**A**) RT-PCR analysis of *SGCB* transcript in patient's derived iPSC before (NT, untreated) and after the delivery of Morpholino 10 µM (MO) documenting the correction of the splicing defect in treated cells. The delivery of a morpholino scrambled sequence (SCR) did not result in splicing correction. A control cell line was also included (CTRL). Bars under the gel represents the proportion of normally spliced (Ex2-Ex3) and abnormally-spliced (Ex2-PE-Ex3) *SGCB* transcripts, as calculated after densitometry. (**B**) Sequence electropherograms displaying the restoration of the physiological splicing after Morpholino delivery.

Gene	Inheritance	OMIM	Locus
ANO5	AD, Ar	166260, 613319, 611307	*11p14.3*
B3GALNT2	Ar	615181	*1q42.3*
B4GAT1	Ar	615287	*11q13.2*
BVES	Ar	616812	*6q21*
CACNA1S	AD	170400, 601887, 188580	*1q32.1*
CAPN3	AD, Ar	618129, 253600	*15q15.1*
CAV3	AD, DD	192600, 123320, 611818, 614321, 606072	*3p25.3*
CLCN1	AD, Ar	160800, 255700	*7q34*
COL6A1	AD, Ar	158810, 254090	*21q22.3*
COL6A2	Ar, AD	255600, 158810, 254090	*21q22.3*
DAG1	Ar	616538, 613818	*3p21.31*
DMD	XLr	300376, 302045, 310200	*Xp21.2-p21.1*
DNAJB6	AD	603511	*7q36.3*
DPM3	Ar	618992, 618992	*1q22*
DYSF	Ar	254130, 253601, 606768	*2p13.2*
FKRP	Ar	613153, 606612, 607155	*19q13.32*
FKTN	Ar	611615, 253800, 613152, 611588	*9q31.2*
GAA	Ar	232300	*17q25.3*
GFPT1	Ar	610542	*2p13.3*
GMPPB	Ar	615350, 615351, 615352	*3p21.31*
POMGNT2	Ar	614830, 618135	*3p22.1*
HNRPDL	AD	609115	*4q21.22*
CRPPA	Ar	614643, 616052	*7p21.2*
KCNJ2	AD	170390, 613980, 609622	*17q24.3*
LAMA2	Ar	607855, 618138	*6q22.33*
LARGE1	Ar	613154, 608840	*22q12.3*
LMNA	AD, Ar	115200, 605588, 181350, 616516, 610140, 176670, 151660, 212112, 248370, 613205, 619793	*1q22*
MYOF	AD	619366	*10q23.33*
PLEC	Ar, AD	616487, 131950, 226670, 612138, 613723	*8q24.3*
POGLUT1	Ar, AD	617232, 615696	*3q13.33*
POMGNT1	Ar	253280, 613151, 613157, 617123	*1p34.1*
POMK	Ar	616094, 615249	*8p11.21*
POMT1	Ar	236670, 613155, 609308	*9q34.13*
POMT2	Ar	613150, 613156, 613158	*14q24.3*
POPDC3	Ar	618848	*6q21*
PYROXD1	Ar	617258	*12p12.1*
RYR1	AD, Ar	117000, 619542, 255320, 145600	*19q13.2*
SCN4A	AD, Ar	170500, 613345, 614198, 608390, 168300	*17q23.3*
SGCA	Ar	608099	*17q21.33*
SGCB	Ar	604286,	*4q12*
SGCD	Ar	606685, 601287	*5q33.2-q33.3*
SGCG	Ar	253700	*13q12.12*
SMPX	XLD, XLR	300066, 301075	*Xp22.12*
TCAP	AD, Ar	607487, 601954	*17q12*
RXYLT1	Ar	615041	*12q14.2*
TNPO3	AD	608423	*7q32.1*
TOR1AIP1	Ar	617072	*1q25.2*

Table S1: Genes included in our NGS-panel.
